# Deep brain stimulation of the superolateral medial forebrain bundle in obsessive-compulsive disorder: Outcomes of symptom domains and their predictive value for treatment efficacy

**DOI:** 10.1016/j.neurot.2026.e01094

**Published:** 2026-10-30

**Authors:** Manuel Czornik, Thomas E. Schlaepfer, Lea Zöllner, Dora M. Meyer-Doll, Selina Rolker, Bastian E.A. Sajonz, Katharina Domschke, Dominique Endres, Kimon Runge, Volker A. Coenen, Juan Carlos Baldermann

**Affiliations:** a Department of Psychiatry and Psychotherapy, Medical Center – University of Freiburg, Faculty of Medicine, University of Freiburg, Freiburg, Germany; b Department of Stereotactic and Functional Neurosurgery, Medical Center of Freiburg University and Medical Faculty of Freiburg University, Freiburg, Germany

**Keywords:** Deep brain stimulation, Obsessive-compulsive disorder, Medial forebrain bundle, Invasive neuromodulation, Predictors of response

## Abstract

Deep Brain Stimulation (DBS) of the superolateral medial forebrain bundle (slMFB) has shown promise in treatment-resistant obsessive-compulsive disorder (OCD). However, little is known about whether specific symptom domains respond better to the intervention or whether they may predict overall treatment outcomes. This study evaluated the effects of slMFB DBS on self-reported obsessive-compulsive symptom domains and investigated whether baseline symptom domains predict clinical response. Fourteen patients with treatment-resistant OCD received slMFB DBS. Symptom severity was assessed with the Obsessive-Compulsive Inventory-Revised (OCI-R) and the Yale-Brown Obsessive-Compulsive Scale at multiple postoperative time points. Linear mixed-effects models examined longitudinal changes, and stepwise multiple regression analyses tested whether baseline symptom proportions predicted treatment response. OCI-R total scores decreased significantly over time (F = 16.80, *p* < .001, *p*FDR = .004), with large effect sizes (*d* = −1.19 at last follow-up). Subscale analyses revealed the greatest improvements in obsessing, checking, and ordering. Regression analyses identified the baseline proportion of obsessing symptoms as a significant predictor of outcome (β = 0.55, *p* = .043, R^2^ = 0.30). slMFB DBS yields substantial improvement in self-reported obsessive-compulsive symptoms, particularly in patients with prominent obsessional features. Obsession-dominant profiles may predict superior response, supporting a phenotype-guided approach to neuromodulation in OCD.

## Introduction

Deep Brain Stimulation (DBS) involves the stereotactic implantation of electrodes into targeted brain regions to modulate dysfunctional neural circuits through continuous electrical stimulation. Initially established in movement disorders, DBS has since been applied to psychiatric conditions, including treatment-resistant obsessive-compulsive disorder (OCD) and treatment-resistant depression (TRD). In OCD, several stimulation targets have been investigated, reflecting the complexity of its underlying neurocircuitry. These include the anterior limb of the internal capsule (ALIC) with the bordering nucleus accumbens (NAc), the ventral capsule/ventral striatum, and the bed nucleus of the stria terminalis (BNST) as well as the subthalamic nucleus [1].

Recently, the superolateral medial forebrain bundle (slMFB) has emerged as a promising novel DBS target for OCD [2,3]. The slMFB is a major fiber tract within the human medial forebrain bundle system, connecting key reward- and affect-related regions, including the NAc, anterior cingulate cortex (ACC), and orbitofrontal cortex (OFC) to the ventral tegmental area (VTA) [4,5]. As a central pathway facilitating dopaminergic signaling, the slMFB plays a vital role in motivation, reinforcement learning, and affect regulation [6]. Given its involvement in reward and motivational circuits, modulation of the slMFB has shown antidepressant effects in TRD [7,8] and improvements in obsessions and compulsions in OCD [2,3,6], highlighting its therapeutic potential.

Neurobiologically, OCD is understood to involve dysfunction within cortico-striato-thalamo-cortical (CSTC) circuits, encompassing brain regions such as the OFC, ACC, striatum (including the NAc and caudate nucleus), and thalamus [9,10]. Hyperactivity or dysregulation in uncertainty-related circuits is believed to underlie the intrusive thoughts and compulsive behaviors that define the disorder [10]. In addition, limbic and reward-related circuits also play a key role [3,4,6], particularly in the motivational and affective domains of OCD [11]. Despite the efficacy of cognitive-behavioral therapy (CBT) and pharmacotherapy with selective serotonin reuptake inhibitors (SSRIs) or clomipramine, approximately 30–40% of patients remain treatment-refractory [12,13]. For these individuals, DBS provides a promising alternative, targeting dysfunctional neural circuits directly.

OCD is a clinically heterogeneous condition, with symptoms spanning multiple domains, including, but not limited to, contamination fears, symmetry concerns, hoarding behaviors, and harm-related obsessions. While patients typically recognize their obsessions as irrational, they feel compelled to engage in compulsive behaviors to reduce distress or prevent perceived harm. Considering the distributed nature of OCD-relevant brain circuits and the symptom heterogeneity [3], it is important to investigate whether slMFB DBS exerts differential effects across specific symptom domains. Identifying baseline symptom subtypes and clinical features that predict DBS response is crucial for personalizing treatment, optimizing patient selection, and reducing unnecessary risks.

This study aims to evaluate the effects of slMFB DBS on distinct obsessive-compulsive symptoms. Programming strategies and stimulation parameters for patients treated with slMFB DBS have been described previously [2,7,14] and were therefore not analyzed in the present study, which focused on symptom-domain changes and baseline predictors of treatment outcome. The present study represents a retrospective secondary analysis of a DBS cohort with substantial overlap with a previously published cohort [2]. Detailed reporting of surgery- and stimulation-related adverse events is available in that publication. No additional serious adverse events were identified during the follow-up period analyzed in the present study. We hypothesize that stimulation of the slMFB will lead to significant reductions in overall symptom severity. Furthermore, we expect differential treatment effects across symptom subtypes, reflecting the slMFB’s connectivity with affective and motivational brain regions. Lastly, we examine whether baseline severity in specific symptom domains predicts therapeutic outcomes, thereby contributing to individualized treatment approaches.

## Materials and Methods

### Study design and participants

This study used a retrospective, within-subject, pre-post design. Data were collected as part of individual open-label therapeutic interventions for treatment-refractory OCD. The primary goal was to assess changes in self-reported and clinician-rated OCD symptom severity before and after DBS of the slMFB. Exploratory analyses investigated whether changes in OCD subdomains varied and whether baseline severity in specific domains predicted the magnitude of clinical response.

Participants were recruited and underwent surgery at the University of Freiburg Medical Center. Patients were either self-referred or referred by their treating psychiatrists. Prior to inclusion, the severity of symptoms and the degree of treatment resistance were evaluated through a series of psychological and medical assessments conducted over multiple appointments. Inclusion criteria for treatment were a diagnosis of severe OCD (F42) and a duration of illness of at least five years. In addition, several adequate treatment attempts must have failed to achieve the desired clinical response: (A) treatment with a selective serotonin reuptake inhibitor (SSRI) at the recommended maximum dosage or maximally tolerated dose for at least 10 weeks (citalopram, fluoxetine, paroxetine 60 mg, fluvoxamine 200 mg, sertraline 200 mg); (B) treatment with a second SSRI at the recommended maximum dosage or maximally tolerated dose for at least 10 weeks; (C) augmentation therapy with lithium, buspirone, or another antipsychotic agent for at least 10 weeks; and (D) psychotherapy (>20 h), specifically CBT including exposure therapy.

Exclusion criteria included the presence of a comorbid psychotic disorder, a clinically significant neurological or medical illness, or a history of cerebral trauma (with the exception of motor tics/Gilles de la Tourette syndrome). Additional exclusion criteria were severe personality disorder, alcohol or substance dependence within the past 12 months, alcohol or substance abuse within the past 6 months, or suicidal ideation/behavior within the past 6 months. Medication adjustments were permitted if clinically indicated and psychotherapy was continued as part of routine clinical care. Part of the present sample (*n* = 8) was previously included in a study by Meyer et al. [2]. However, the current study addresses different research questions and includes additional data, outcome measures, and analyses that were not reported previously.

The severity of patients’ symptoms was assessed at multiple time points both before and after surgery, and the assessments were grouped into different time bins (T1 = baseline (up to 6 months preoperatively); T2 = 3–9 months postoperatively; T3 = 10–16 months postoperatively). If a patient had multiple psychological assessments within a single time bin, their mean value was used. The analysis of clinical data was approved by the local ethics committee within a retrospective and prospective DBS registry framework (Nr. 21–1274_2). All participants were informed about the procedure, potential risks, and the scientific use of their data, and provided written informed consent. All patients had received slMFB DBS with electrode position targeting the VTA (slMFB midbrain target).

### Assessment instruments

The Obsessive-Compulsive Inventory-Revised (OCI-R) is a self-report measure assessing the severity of obsessive-compulsive symptoms across six symptom domains. It is a shortened 18-item version of the original 42-item OCI and captures six symptom domains: washing, hoarding, neutralizing, obsessing, ordering, and checking. Developed by Foa et al. [15], the OCI-R has demonstrated high internal consistency (α = 0.81) and good test-retest reliability. The German version validated by Gönner et al. [16] provides a psychometrically robust tool for assessing OCD symptoms in both clinical and nonclinical populations. Although the OCI-R was not specifically designed as a comprehensive assessment of OCD symptom domains, its six subscales have demonstrated good factorial validity and are commonly used to characterize the relative prominence of OCD symptom domains [17,18]. In the present study, these subscales were used to examine domain-specific changes in symptoms over time and their association with treatment outcome, rather than to establish definitive or discrete OCD symptom domains. The OCI-R served as the primary outcome measure, providing both domain-specific and total symptom severity scores for quantitative analyses.

The Yale-Brown Obsessive-Compulsive Scale (Y-BOCS) is a semi-structured clinician-rated interview assessing the overall severity of obsessions and compulsions, independent of symptom type [19,20]. It consists of 10 items rated on a 5-point Likert scale (total range: 0–40) and is widely regarded as the clinical gold standard for OCD severity assessment. The Y-BOCS provides a total severity score as well as separate subscores for obsession and compulsion severity. Importantly, it is not intended to classify patients according to distinct OCD subtypes, but rather to quantify symptom severity across different manifestations of OCD. In this study, it was used both to characterize baseline clinical severity and to quantify overall treatment-related change and obsession and compulsion subscores. Since it is the more widely established instrument for assessing treatment response in clinical trials, changes in Y-BOCS scores were used as the dependent variable in linear regression analyses to examine potential predictors of overall slMFB DBS efficacy.

### Statistical analyses

All statistical analyses were conducted using IBM SPSS Statistics version 31 (IBM Corp., Armonk, NY, USA). Longitudinal changes in obsessive-compulsive symptoms following DBS were analyzed using linear mixed-effects models (LMMs). Separate models were fitted for the OCI-R total score and each OCI-R subscale score as dependent variables. Time was included as a fixed effect and was categorized into three assessment periods (T1, T2, and T3). Repeated measurements were modeled within subjects, with an unstructured covariance matrix specified to account for correlations between repeated observations. A random intercept was included for each participant to account for inter-individual differences in baseline symptom severity. When multiple assessments were available within the same time period, all scores were averaged to obtain a single value for that interval. Model parameters were estimated using restricted maximum likelihood (REML). Model assumptions were evaluated by visual inspection of residual plots and normal Q-Q plots to assess homoscedasticity and normality of residuals. The significance of the fixed effect of time was evaluated using two-tailed tests with an alpha level of 0.05. Effect sizes (Cohen’s *d*) were calculated from the estimated marginal means and corresponding standard errors obtained from the LMMs. To account for multiple testing, *p*-values were adjusted using the Benjamini–Hochberg false discovery rate (FDR) procedure [21]. Statistical significance was defined as a two-tailed adjusted *p*-value <.05. The LME approach was chosen for its ability to model repeated measures within subjects and handle smaller clinical samples.

As a sensitivity analysis, a baseline-adjusted LME was performed to account for differences in initial severity across OCI-R subscales. To do so, the subscale scores were modeled jointly, with time, subscale, and their interaction as fixed effects and the corresponding baseline subscale score as a covariate. Baseline-adjusted estimated marginal means were calculated to descriptively compare symptom severity across subscales at follow-up.

In addition, differential longitudinal changes in obsessions and compulsions were examined using the Y-BOCS total score and the obsession and compulsion subscores separately. Additionally, we calculated a baseline-adjusted LME to account for differences in baseline severity across Y-BOCS subdomains (i.e. obsession and compulsion subscores). All model estimation and statistical inferences involving the Y-BOCS followed the same procedure as analysis of the OCI-R.

To explore predictors of treatment response, a stepwise multiple linear regression was conducted, with baseline OCI-R subscale proportions (i.e., each subscale expressed as the ratio of its score to the total OCI-R score) entered as predictor variables, and percentage change in Y-BOCS total score as the dependent variable. This approach allowed evaluation of the relative contribution of each symptom domain, independent of overall OCD severity in the respective subdomain at baseline.

Last, to test whether changes in obsessive-compulsive symptoms (as assessed with the Y-BOCS) correspond to changes in depression scores (as assessed with the MADRS), we correlated absolute change scores for both follow-ups (T2 and T3) using Spearman Correlations.

## Results

### Patient characteristics and demographics

Data from 15 patients treated between 2017 and 2024 were initially considered. One patient was excluded due to missing data in the predefined assessment intervals, resulting in a final sample of 14 patients (6 female, 8 male). The mean age of the final sample was 42.9 years (*SD* = 11.0; range = 25–59 years). The mean duration of illness between initial diagnosis and surgery was 18.1 years (*SD* = 10.7). Before surgery, the mean symptom severity was 33.0 (*SD* = 14.2) on the OCI-R and 30.4 (*SD* = 5.6) on the Y-BOCS. All patients also showed clinically relevant depressive symptoms at baseline, with a mean BDI-II score of 31.4 (*SD* = 9.3) and a mean MADRS score of 20.1 (*SD* = 8.6). Additional psychiatric comorbidities included mild to moderate personality disorders (*n* = 4), anxiety disorders (*n* = 3), post-traumatic stress disorder (n = 2), eating disorders (*n* = 2), and attention-deficit/hyperactivity disorder (*n* = 2). Two patients had a history of alcohol and/or benzodiazepine dependence, and one patient had bipolar disorder.

### Changes in OCI-R total and Y-BOCS

To examine longitudinal changes in obsessive-compulsive symptom severity, a linear mixed-effects model was conducted across three time points (T1, T2, and T3) for the OCI-R total (Fig. 1A) and subscale scores (Fig. 1B). The overall OCI-R total score showed a significant main effect of time (*F* = 16.80, *p* < .001), indicating a robust reduction in self-reported OCD symptoms. Estimated marginal means decreased from *M* = 33.04 (T1) to *M* = 20.16 (T2) and *M* = 17.72 (T3). This effect remained significant following FDR correction (Benjamini-Hochberg, *p*FDR = .004). Corresponding FDR-adjusted *p*-values are presented in Table 1. Large effect sizes were observed for both T1–T2 (*d* = −0.87) and T1–T3 (*d* = −1.19), supporting the clinical relevance of the improvement. For the clinician-rated Y-BOCS total, a significant effect of time was also detected (*F* = 46.45, *p* < .001), with estimated marginal means decreasing from *M* = 30.44 (T1) to *M* = 16.42 (T2) and *M* = 14.32 (T3).

**Fig. 1:**
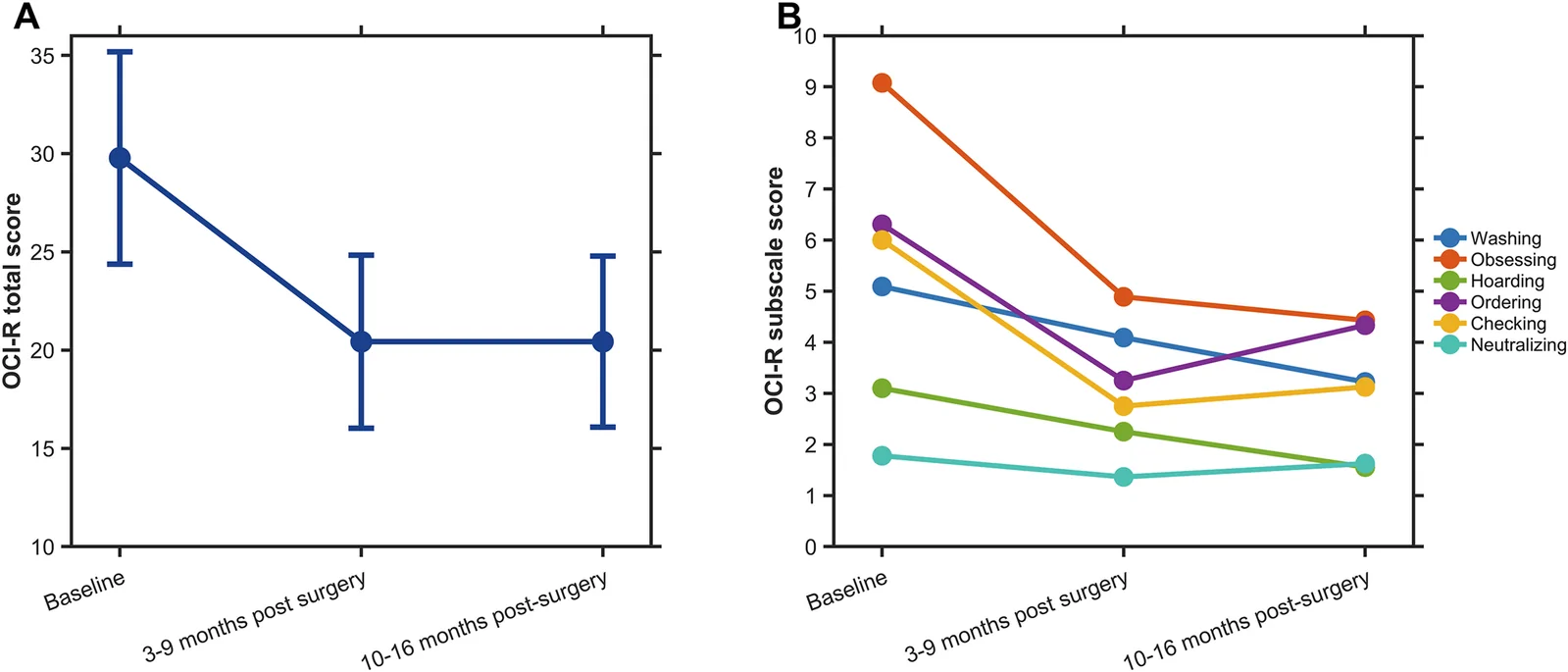
Changes in the Obsessive-Compulsive Inventory Revised (OCI-R) during Deep Brain Stimulation A. Longitudinal changes in averaged OCI-R total scores for patients with obsessive-compulsive disorder (*n* = 14), including ±1 standard error across three time points. B. Longitudinal changes in mean OCI-R subscale scores.

**Table 1:** False Discovery Rate (FDR) corrected *p*-values of the OCI-R total and subscales.

Rank	Scale	FDR-adjusted *p*	*d* (T1–T2)	*d* (T1–T3)
1	Obsessing	.004∗∗	−1.37	−1.80
2	OCI-R total	.004∗∗	−0.87	−1.19
3	Ordering	.014∗	−0.64	−0.53
4	Checking	.025∗	−0.64	−0.84
5	Neutralizing	.033∗	−0.37	−0.40
6	Hoarding	.033∗	−0.13	−0.56
7	Washing	.041∗	−0.25	−0.39

FDR = False Discovery Rate; OCI-R = Obsessive-Compulsive Inventory-Revised. ∗*p* < .05, ∗∗*p* < .01.

### Changes in OCI-R- and Y-BOCS subscales

Analyses of the OCI-R subscales revealed varying magnitudes of change over time (see Table 1 for detailed statistics). Washing showed a significant main effect of time (*F* = 4.27, *p* = .041, *p*FDR = .041), with mean scores decreasing from 5.46 to 3.55 and small-to-moderate effect sizes (*d* = −0.25 to −0.39). Obsessin*g* exhibited the most pronounced reduction (*F* = 15.38, *p* < .001, *p*FDR = .004), with scores declining from 9.25 to 4.37 and very large effect sizes (*d* = −1.37 to −1.80). Hoarding demonstrated moderate improvements (*F* = 4.92, *p* = .028, *p*FDR = .033), accompanied by effect sizes increasing from *d* = −0.13 to −0.56. Ordering also showed a significant decrease over time (*F* = 8.50, *p* = .006, *p*FDR = .014), with moderate effects (*d* = −0.64 to −0.53). Checking declined significantly (*F* = 6.63, *p* = .014, *p*FDR = .025), characterized by small-to-moderate effects (*d* = −0.64 to −0.84). Finally, neutralizing displayed a modest but statistically significant reduction (*F* = 5.11, *p* = .027, *p*FDR = .033), with effect sizes ranging from *d* = −0.37 to −0.40. Overall, the largest effects were observed for obsessing, followed by checking and ordering, whereas neutralizing, hoarding, and washing showed comparatively smaller changes. This pattern is consistent with the mixed-model results, indicating substantial reductions in obsessional symptoms and moderate improvements across other symptom domains following slMFB stimulation. The baseline-adjusted sensitivity analysis showed that scores at T2 and T3 were strongly associated with baseline scores (*F* = 217.06, *p* < .001). Additionally, baseline-adjusted scores at T2 and T3 were differentially reduced across subscales (*F* = 2.58, *p* = .030). The main effect of time (*F* = 1.77, *p* = .186) and the time × subscale interaction (*F* = 0.39, *p* = .857) were not significant, indicating that OCI-R scores did not differ significantly between T2 and T3 and that changes from T2 to T3 did not differ significantly between subscales. After adjustment for baseline subscale severity, obsessing showed the numerically lowest estimated marginal mean at both T2 and T3 (see Supplemental Table 1), indicating that the pattern of pronounced improvement in obsessing symptoms persisted after accounting for differences in initial symptom severity across domains.

When analyzing the subscales of the Y-BOCS separately, we observed a significant effect of time on both obsessions (*F* = 40.93, *p* < .001), and compulsions (*F* = 18.56, *p* < .001). Estimated marginal means indicated a reduction in obsession severity from *M* = 17.06 at baseline (T1) to *M* = 8.82 (T2), and *M* = 7.57 (T3). Similarly, compulsion severity decreased from *M* = 13.38 at T1 to *M* = 7.80 at T2, and *M* = 7.18 at T3. After adjustment for baseline subscale severity, we found a significant effect of baseline score (*F* = 59.76, *p <* .001) but not of subscale (*F* = 1.92, *p* = .190). Numerically, obsessions showed the lowest marginal means at both T1 and T2 (see Supplemental Table 2), indicating that obsessions were more pronouncedly reduced than compulsions).

### Predictors of treatment response

A multiple linear regression was conducted to determine which OCI-R subtypes predicted treatment response, using baseline OCI-R subscale proportions (i.e., each subscale expressed as a ratio of the individual’s total OCI-R score) as predictor variables. Using a stepwise approach, only the obsessing subscale emerged as a significant predictor (β = 0.55, *p* = .043), indicating that a higher relative contribution of obsessional symptoms at baseline predicted greater improvement in Y-BOCS scores at long-term follow-up. The final model explained approximately 30% of the variance in treatment response (R^2^ = 0.300). Other subscales – including checking, ordering, neutralizing, washing, and hoarding – did not reach statistical significance and thus did not contribute meaningfully to the model. Fig. 2a displays the standardized beta coefficients (β) for all subscales, while Fig. 2b illustrates the linear relationship between baseline proportional obsessing scores and long-term change in Y-BOCS total scores for individual patients. Although not statistically significant, negative beta values for hoarding and washing suggested a trend toward weaker treatment response when these domains dominated prior to surgery.

**Fig. 2:**
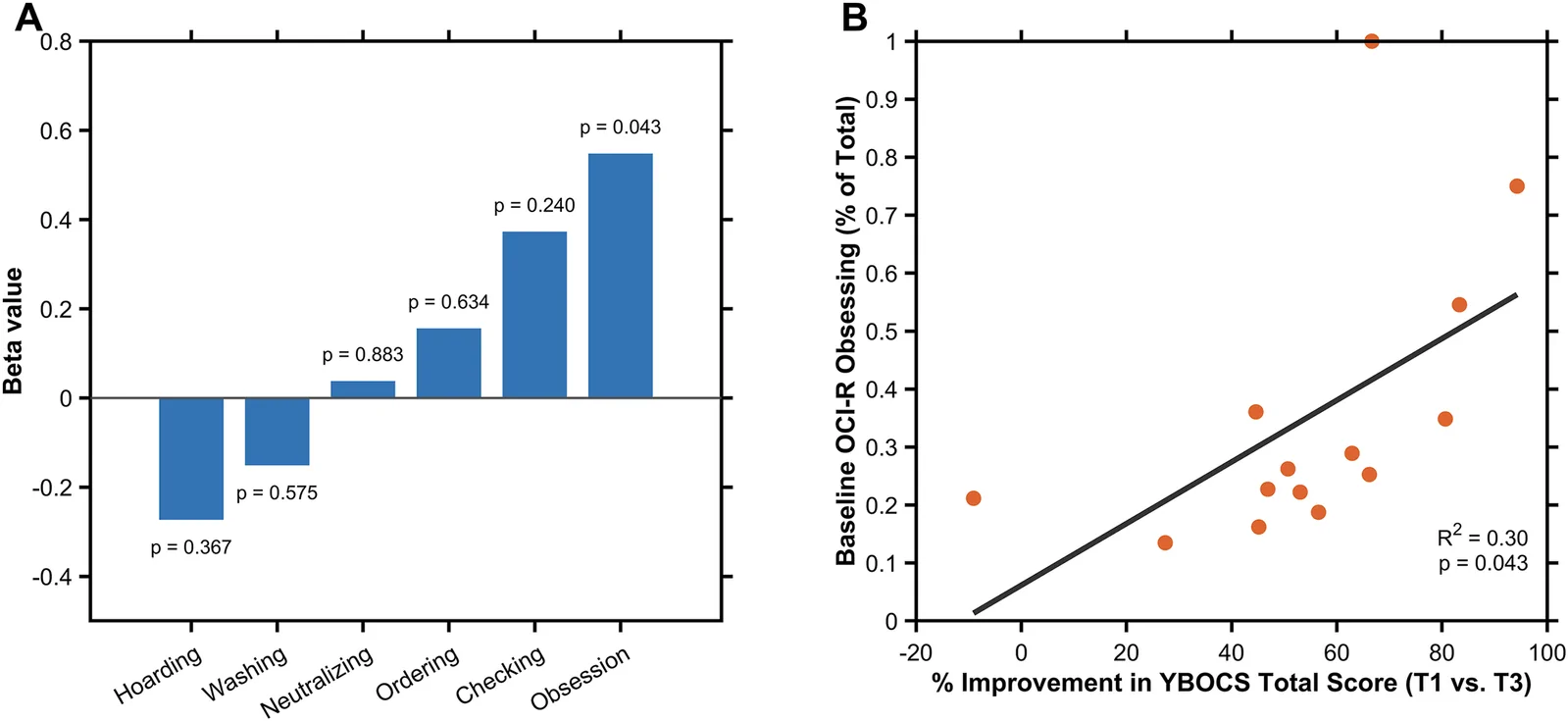
Prediction of treatment response from baseline symptom subdomains A. Standardized beta coefficients (β) for Obsessive-Compulsive Inventory-Revised (OCI-R) subscales predicting treatment response in the Yale-Brown Obsessive-Compulsive Scale (Y-BOCS) at long-term follow-up. B. Relationship between baseline proportional OCI-R obsessing scores and long-term improvement in Y-BOCS total scores following slMFB stimulation. Each point represents an individual patient. The x-axis shows the change in Y-BOCS from pre-operative baseline (T1) to long-term follow-up (T3), with positive values indicating symptom improvement. The y-axis represents the proportion of obsessing symptoms relative to the total OCI-R score at baseline.

### Association between changes in obsessive-compulsive and depressive symptoms

To examine whether improvement in obsessive-compulsive symptoms was associated with concurrent improvement in depressive symptoms, correlations were calculated between changes in Y-BOCS and MADRS scores from T1 to T2 and from T1 to T3. No significant associations were observed at either interval (T1–T2: *r* = 0.12, *p* = .713; T1–T3: *r* = 0.23, *p* = .438).

## Discussion

The present study found pronounced reductions in self-reported obsessive-compulsive symptoms following DBS of the slMFB (VTA) in patients with treatment-refractory OCD. Across the sample, total OCI-R scores declined substantially, and effect sizes indicated a large overall improvement. However, the pattern of response across the six OCI-R subdomains revealed notable heterogeneity, indicating that slMFB stimulation does not affect all OCD symptom domains equally. The most pronounced improvements were observed for the obsessing subscale, followed by checking, ordering, and neutralizing, while washing as well as hoarding showed comparatively smaller effects. Importantly, the preoperative distribution of symptom burden across subdomains predicted overall treatment response. In particular, a higher relative load of obsessions was associated with a greater postoperative reduction in overall OCD severity. This differential response pattern and its potential to predict subsequent treatment response at baseline suggests that the therapeutic effects of slMFB DBS may depend on the dominant neurocircuitry [3] underlying each specific symptom domain.

DBS is an established treatment option for individuals with severe, treatment-resistant OCD [22]. Within the different brain regions that have been targeted with DBS for OCD, the slMFB has recently emerged as a promising target [2]. As a main glutamatergic feedback loop to the VTA/midbrain, it has been targeted in the VTA or in ALIC [3,23,24]. The mean symptom reduction in the current analysis of approximately 53% in the overall self-reported OCI-R score aligns with prior reports of clinically significant benefit in clinician-rated assessments, often accompanied by improvements in mood, motivation, and quality of life [2,6]. Our analysis adds a patient-centered perspective, capturing subjective symptom changes that complement clinician-rated outcomes and reflect the patients’ own experience of therapeutic benefit. Critically, the current investigation suggests that symptom domains may differ in their degree of improvement following slMFB DBS.

Our analysis showed that reductions in OCI-R scores after slMFB-DBS differed across subdomains, even after correcting for the respective baseline values. Among all subdomains, the obsession subscore showed the most substantial improvement even when correcting for baseline severity in each subdomain. Regression analyses further identified baseline obsessing severity as the only significant predictor of subsequent clinical improvement. Specifically, patients with a higher relative proportion of obsessional symptoms at baseline exhibited greater reductions in Y-BOCS scores, accounting for 30% of the variance in treatment outcome. This suggests that individuals whose OCD is dominated by affectively charged intrusive thoughts – rather than purely behavioral compulsions – may derive the greatest benefit from slMFB DBS [3]. To our knowledge, this observation has not been reported for other DBS targets in OCD. Thus, we argue that this finding might relate to the specific neurobiological implications of the slMFB/VTA target.

From a neurobiological perspective, the slMFB is a key component of the brain’s reward and motivation circuitry, connecting the VTA with dopaminergic projection sites such as the NAc, OFC, and ACC [6]. Modulation of this pathway likely normalizes dysregulated reward processing and affective reactivity, both of which are implicated in the maintenance of obsessive thoughts and affective distress. The prominent reduction in obsessing symptoms is consistent with this proposed mechanism, as intrusive thoughts in OCD are often perpetuated by heightened negative affect and dysfunctional appraisal of internal experiences. By improving affect regulation and motivational drive, slMFB stimulation may attenuate the emotional salience of obsessive cognitions, reducing both their frequency and distressing quality. We have previously speculated about a potential role of Brodman’s area 8 M, which is involved in the resolution of uncertainty and strongly connected to the VTA via the slMFB [3].

Moreover, this interpretation is consistent with previous work by Liebrand et al. [25], who found that stimulation along the anterior limbic radiation near the slMFB was associated with superior outcomes compared to stimulation near the anterior thalamic radiation, likely due to concurrent improvements in mood and anxiety regulation. Given the pronounced antidepressant effects of slMFB DBS in major depressive disorder [8,14], these findings suggest shared neurobiological substrates between depressive and obsessive ruminations — both characterized by repetitive, affect-laden thought loops mediated through fronto-limbic circuits.

Since all participants showed clinically relevant depressive symptoms, it is conceivable that improvement in mood contributed to the observed reduction in OCD symptoms. To explore this possibility, we examined the association between changes in Y-BOCS and MADRS scores. No significant correlations were observed at either postoperative interval, suggesting that improvement in OCD symptoms was not closely associated with the magnitude of antidepressant response in this cohort. Nevertheless, given the small sample size, this exploratory analysis should be interpreted with caution and cannot exclude a partial contribution of mood improvement to the observed anti-obsessional effects.

Beyond the large clinical effects observed in the obsessing subscale, checking, ordering, and neutralizing showed more moderate improvements while hoarding and washing displayed the smallest improvements. These findings partially align with the observations of Denys et al. [26] which is not surprising given that ALIC DBS recruits the slMFB as a pathway from further proximal [23]. Thavarajasingam et al. [27] reported a clear association between hoarding symptoms and poor DBS response in other target regions and further suggested that symptom dimensions such as symmetry, hoarding, and perfectionism predict poorer outcomes due to underlying sensorimotor circuit dysfunctions. Welter et al. [28] described hoarding as resistant to DBS, suggesting that hoarding-dominant OCD may require targeting different brain regions. Interestingly, although hoarding symptoms improved significantly by self-report, baseline hoarding severity predicted a negative response trend. The observation that hoarding symptoms have consistently been associated with poorer outcomes than other OCD symptoms does not only apply to DBS, but was also shown for other pharmacological or behavioral treatments [29]. These symptoms often involve habitual, ritualized behaviors that may be less influenced by emotional evaluation. The slMFB’s reward-related modulation likely only partially affects these subtypes. However, as outlined, this relationship may also be the case for other DBS targets.

Interestingly, the preferential improvement in obsessional symptoms observed in our cohort may also be relevant to the ongoing discussion regarding the functional distinction between slMFB and anteromedial STN (amSTN) DBS. Although these targets are anatomically adjacent, we have previously shown that both target regions stimulate both overlapping but also distinct neural circuits [3]. Our current results further strengthen the notion that both target regions may result in different clinical outcome profiles. Mallet et al. [30] reported larger improvements in compulsive symptoms following amSTN DBS, whereas our results suggest that slMFB has larger effects on obsessions compared to compulsions. Further, Tyagi et al. [31] found that amSTN DBS had greater effects on cognitive flexibility and less so on mood compared to ALIC DBS. Since slMFB DBS appears to have predominantly affective and motivational effects, this is a further sign that slMFB DBS and amSTN DBS can be separated both clinically and regarding their connectivity profiles [3]. However, these comparisons are still largely indirect, and whether slMFB and amSTN represent functionally distinct targets or different access points to a common therapeutic network remains to be determined in direct comparative studies.

Our data support a growing emphasis on phenotype-guided neuromodulation, where stimulation targets are selected based on predominant symptom domains and associated circuit dysfunctions. Such an approach has already been established in DBS for Parkinson’s disease, where multiple target regions are available, and clinical decision-making is based on individual symptoms and the respective optimal stimulation site. In the sense of precision psychiatry, our findings suggest that a similar approach may be adaptable to neuromodulation of OCD. This is even more important, given the heterogeneity of OCD, as a “one-target-fits-all” model is unlikely to achieve optimal outcomes. Future research may focus on deep phenotyping, by integrating neuroimaging-based tractography, electrophysiological markers, (epi)genetic markers, and neuropsychological symptoms to identify individualized targets within the broader reward-limbic-cognitive control network [[32], [33], [34], [35], [36], [37]]. Through such individualized approaches, DBS has the potential to advance precision medicine in psychiatry and substantially improve the quality of life for patients with otherwise intractable OCD.

Our results should be interpreted in light of several limitations: The study’s retrospective design and small sample size limit generalizability and preclude causal inference. Furthermore, the absence of a control group and sham stimulation condition means that the observed symptom improvements cannot be unequivocally attributed to DBS alone. Potential contributions of nonspecific factors, including placebo effects, expectation effects, natural symptom fluctuations, and concomitant treatments, cannot be fully excluded. Stimulation parameters were individually optimized in clinical practice, introducing variability that may further explain differential effects across symptom domains. Furthermore, reliance on self-report measures for some analyses may have introduced subjective bias – on the other hand, clinician-rated scales may introduce similar issues from the clinician’s perspective. In addition, symptom-domain analyses were based on OCI-R subscales rather than a dedicated dimensional measure of OCD. The higher baseline severity of obsessions may have introduced a potential regression-to-the-mean effect. However, a sensitivity analysis including baseline Y-BOCS severity as an additional covariate yielded results consistent with the primary analysis, with obsessions showing the most robust decrease over time. Prospective trials with larger samples and appropriate comparison conditions are needed to validate these preliminary findings and to refine predictors of response.

In conclusion, DBS of the superolateral medial forebrain bundle was associated with substantial and clinically meaningful reductions in self-reported obsessive-compulsive symptoms, particularly in patients with prominent obsessional and affectively driven features. The differential response across symptom domains reinforces the heterogeneity of OCD and highlights the value of tailoring neuromodulatory interventions to individual symptom profiles. Baseline severity of obsessional symptoms emerged as the strongest predictor of outcome, suggesting that slMFB stimulation may be most effective for OCD subtypes characterized by intrusive thoughts and affective dysregulation. Together, these findings position the slMFB as a promising target within a phenotype-informed framework of DBS for OCD, moving the field further toward precision neuromodulation.

## Author contributions

Conceptualization: JCB, MC, LZ, TES. Data curation: MC, DM-D. Formal analysis: LZ, MC, JCB, DM-D. Investigation: JCB, MC, VAC, TES, DM-D, BEAS, SR. Methodology: JCB, LZ, MC, TES, VAC. Project administration: JCB, TES, VAC, MC, DM-D. Resources: TES, VAC, KD, JCB. Supervision: JCB, MC, TES, VAC. Validation: JCB, MC, DM-D. Visualization: JCB. Writing—original draft: MC, LZ, JCB, VAC. Writing—review and editing: MC, TES, LZ, DM-D, SR, BEAS, KD, DE, KR, VAC, JCB.

## Declaration of competing interest

The authors declare that they have no known competing financial interests or personal relationships that could have appeared to influence the work reported in this paper.The authors declare the following financial interests/personal relationships which may be considered as potential competing interests: Volker A. Coenen reports a relationship with Brainlab SE that includes: funding grants. Volker A. Coenen reports a relationship with CereGate GmbH that includes: consulting or advisory. Volker A. Coenen reports a relationship with CorTec GmbH that includes: consulting or advisory. Volker A. Coenen reports a relationship with Inbrain that includes: consulting or advisory. Volker A. Coenen reports a relationship with Boston Scientific Corporation that includes: funding grants, speaking and lecture fees, and travel reimbursement. Juan Carlos Baldermann reports a relationship with Boston Scientific Corporation that includes: funding grants. Dora M. Meyer-Doll reports a relationship with Evangelisches Studienwerk e.V. that includes: funding grants. Dora M. Meyer-Doll reports a relationship with Wissenschaftliche Gesellschaft Freiburg im Breisgau that includes: funding grants. Katharina Domschke reports a relationship with Janssen-Cilag GmbH that includes: speaking and lecture fees. Katharina Domschke reports a relationship with Neurotorium that includes: board membership. Katharina Domschke reports a relationship with Lundbeck Foundation that includes: board membership. Bastian E. A. Sajonz reports a relationship with Precisis that includes: consulting or advisory. Bastian E. A. Sajonz reports a relationship with Ceregate that includes: funding grants. Bastian E. A. Sajonz reports a relationship with Boston Scientific Corporation that includes: travel reimbursement. Bastian E. A. Sajonz reports a relationship with Medtronic PLC that includes: speaking and lecture fees and travel reimbursement. Bastian E. A. Sajonz reports a relationship with Insightec that includes: speaking and lecture fees and travel reimbursement. Volker A. Coenen has patent #63/253,740 pending to University of Freiburg. Thomas E. Schlaepfer has patent #63/253,740 pending to University of Freiburg. If there are other authors, they declare that they have no known competing financial interests or personal relationships that could have appeared to influence the work reported in this paper.
