# Supplementary material for: Deep brain stimulation of the superolateral medial forebrain bundle in obsessive-compulsive disorder: Outcomes of symptom domains and their predictive value for treatment efficacy
Source: Neurotherapeutics. 2026 Oct 30;24(1):e01094. doi: 10.1016/j.neurot.2026.e01094 (PMC13743482; doi:10.1016/j.neurot.2026.e01094)
Supplement: Supplementary file 1 [file mmc1.docx]

**Supplementary Information**

**Supplemental Table 1.** Baseline-Adjusted Estimated Marginal Means of OCI-R Subscale Scores at Follow-Up

| Timepoint (T) and OCI-R Subdomain | | Mean | Std. Error | 95% Confidence Interval | |
| --- | --- | --- | --- | --- | --- |
|  |  |  |  | Lower Bound | Upper Bound |
| 2 | Checking | 3.041^b^ | 0.572 | 1.898 | 4.184 |
|  | Hoarding | 4.177^b^ | 0.576 | 3.026 | 5.329 |
|  | Neutralizing | 3.790^b^ | 0.589 | 2.614 | 4.965 |
|  | Obsessing | 2.845^b^ | 0.588 | 1.671 | 4.018 |
|  | Ordering | 3.073^b^ | 0.570 | 1.933 | 4.213 |
|  | Washing | 4.100^b^ | 0.569 | 2.962 | 5.237 |
| 3 | Checking | 2,578^b^ | 0.632 | 1.320 | 3.836 |
|  | Hoarding | 3.268^b^ | 0.641 | 1.993 | 4.544 |
|  | Neutralizing | 3.462^b^ | 0.644 | 2.181 | 4.744 |
|  | Obsessing | 1.960^b^ | 0.653 | 0.660 | 3.259 |
|  | Ordering | 3.203^b^ | 0.634 | 1.941 | 4.465 |
|  | Washing | 4.133^b^ | 0.631 | 2.876 | 5.390 |

OCI-R = Obsessive-Compulsive Inventory-Revised.

**Supplemental Table 2.** Baseline-Adjusted Estimated Marginal Means of Y-BOCS Subscale Scores at Follow-Up

| Timepoint (T) and Y-BOCS Subdomain | | Mean | Std. Error | 95% Confidence Interval | |
| --- | --- | --- | --- | --- | --- |
|  |  |  |  | Lower Bound | Upper Bound |
| 2 | Obsessions | 8.157^b^ | 0.907 | 6.202 | 10.112 |
|  | Compulsions | 8.771^b^ | 0.655 | 7.350 | 10.191 |
| 3 | Obsessions | 6.788^b^ | 1.115 | 4.383 | 9.194 |
|  | Compulsions | 7.968^b^ | 1.059 | 5.684 | 10.253 |

Y-BOCS = Yale-Brown Obsessive-Compulsive Scale.
